# The complete chloroplast genome sequence of *Turpinia montana* (Staphyleaceae)

**DOI:** 10.1080/23802359.2020.1821821

**Published:** 2020-09-21

**Authors:** Wei-Hong Sun, Yi-Fan Wang, De-Qiang Chen, Shuang-Quan Zou

**Affiliations:** a College of Forestry, Fujian Agriculture and Forestry University, Fuzhou, P. R. China; b Fujian Colleges and Universities Engineering Research Institute of Conservation and Utilization of Natural Bioresources, College of Forestry, Fujian Agriculture and Forestry University, Fuzhou, P. R. China; c Key Laboratory of National Forestry and Grassland Administration for Orchid Conservation and Utilization at College of Forestry, Fujian Agriculture and Forestry University, Fuzhou, P. R. China

**Keywords:** Rosids, *Turpinia montana*, plastid genome, phylogeny

## Abstract

The Rosids are characterized by remarkable morphological and ecological diversity. Here, we provide the completed plasmid genome of *Turpinia montana.* The complete chloroplast size of *T. montana* is 160,111 bp, including a large single-copy (LSC) region of 89,631 bp, a small single-copy (SSC) region of 18,247 bp, a pair of invert repeats (IR) regions of 26,120 bp. Plastid genome contains 131 genes, 86 protein-coding genes, 37 *tRNA* genes, and eight *rRNA* genes. Phylogenetic analysis base on 23 plastid genomes indicates that *T. montanas* is clustered with the plants of the Euscaphis japonica and *Staphylea bumalda*.

The Rosids are characterized by remarkable morphological and ecological diversity, with a total of 17 orders, containing 140 families and about 70,000 species (APG II 2003; APG III 2009; APG IV 2016). Their life forms include herbs, trees, aquatic plants, and succulents. Some of them are important economic crops (e.g. Rosaceae, Fabaceae, and Brassicaceae), and others are important trees (e.g. Betulaceae, Sapindaceae, and Fagaceae). The Rosids mainly divided into Fabids and Malvids, among which Fabids are divided into COM clade (Celastrales, Oxalidales, and Malpighiales) and the nitrogen-fixing clade (Cucurbitales, Fagales, Fabales, and Rosales; Qiu et al. 2010; APG IV 2016). Although many species of Rosids have completed chloroplast sequencing, the phylogenetic positions of the COM branch, Nitrogen-fixing branch, and the Malvids branch are still controversial (Zhao et al. 2016). The basal group is an ideal group for study the origin and evolution of species taxa. Here, we report the complete plastid genome of *Turpinia montana*, a species of the base taxa of the Malvids. *Turpinia montana* mainly distributed in South of China, Java and Sumatra in Indonesia (Li et al. 2008). The leaves and branches of *T. montana* are rich in flavonoids, triterpenes, and other secondary metabolites, which are widely used in medicinal materials (Lei et al. 2019; Xiao et al. 2019). Turs, the complete plastid genome of *T. montana* will be helpful to study the origin, evolution, and diversification within the Rosids and provide theoretical basis for effective conservation strategies for this important plant.

Since the leaves of *T. montana* are rich in secondary metabolism, we collected freshly germinated leaves in Qingyun Mountain, Yongtai County, Fuzhou City, Fujian province, China (118°57′37′′E, 25°46′47′′N) for total genomic DNA extraction. The voucher specimens of branches, leaves, flowers, and fruits are kept in the Herbarium of the College of Forestry, Fujian Agriculture, and Forestry University (specimen code FAFU08031). A modified acetyl trimethyl ammonium bromide method was used to extract genomic DNA (Porebski et al. 1997) from *T. montana* leaves. The concentration and quality of the recovered DNA were measured by NanoDropTM spectrophotometry (Thermo) at 260 and 280 nm, and the purity of the DNA was verified by 0.8% (w/v) agarose gel. The high-quality DNA was randomly interrupted 400 bp using the Covaris ultrasonic breaker to construct a library. Repair the end of the small fragment, and then add an A to the end of the small fragment 3′ to connect to the adapter because there is a T at the adapter 3′end. PCR enriches the target fragments, and finally detects the quality of the library. The library was sequenced on Illumina Hiseq Xten platform for PE150, generating about approximately 6.89 GB of raw data. The raw data was filtered by script in the cluster with default parameters of − l5, −p0.5, −n 0.1. With *Staphylea trifolia* complete plastid genome (GeneBank accession: MK488092) as reference, we assembled the plastid genome of *T. montana* by using GetOrganelle pipe-line (https://github.com/Kinggerm/GetOrganelle; Jin et al. 2019). The Bandage (Wick et al. 2015) was used to view and edit assembled sequences, and the Geneious version 11.1.5 (America) was used to annotation the plastid genome of *T. montana* (Kearse et al. 2012). Finally, the annotation result was visualized by using the online tool OGDRAW (http://ogdraw.mpimp-golm.mpg.de/; Lohse et al. 2013). The complete plastid genome sequence of *T. montana* has been submitted to GenBank with the accession number MT501463.

The complete plastid genome sequence length of *T. montana* was 160,111 bp, in which a pair of inverted repeats (IR) regions were 26,120 bp, each, a large single-copy (LSC) region was 89,631 bp, and small single-copy (SSC) region was 18,247 bp. The complete genome GC content was 37.4%, and the complete plastid genome encodes 131 genes, containing 86 protein-coding genes, 37 transfer *RNA* (tRNA) genes, and eight ribosomal (*rRNA*) genes. To reveal the phylogenetic position within the Rosids, a phylogenetic tree was conducted based on complete plastid genomes of 23 species, with two monocotyledons, *Dendrobium chrysanthum* and *Dendrobium brymerianum*, as the outer group. These genome data were downloaded from NCBI GenBank (https://www.ncbi.nlm.nih.gov/search/all/?term=GenBank). The chloroplast genome of *T. montanas* was aligned with other 22 chloroplast genome sequence, using RAxML to construct the maximum likelihood tree (Stamatakis 2014). The phylogenetic tree showed that the Staphyleaceae species *T. montanas*, *Euscaphis japonica*, and *Staphylea bumalda* cluster together (Xiang et al. 2019; Jing-Yao et al. 2020), but they are not the base group of the Malvids. This result is inconsistent with the results of previous studies that *T. montana* is located at the base of Malvids (APG IV 2016). The completed plastid genome sequence of *T. montana* can help reveal its phylogenetic position, and provide data for future conservation efforts and biological research (Figure 1).

**Figure 1.**
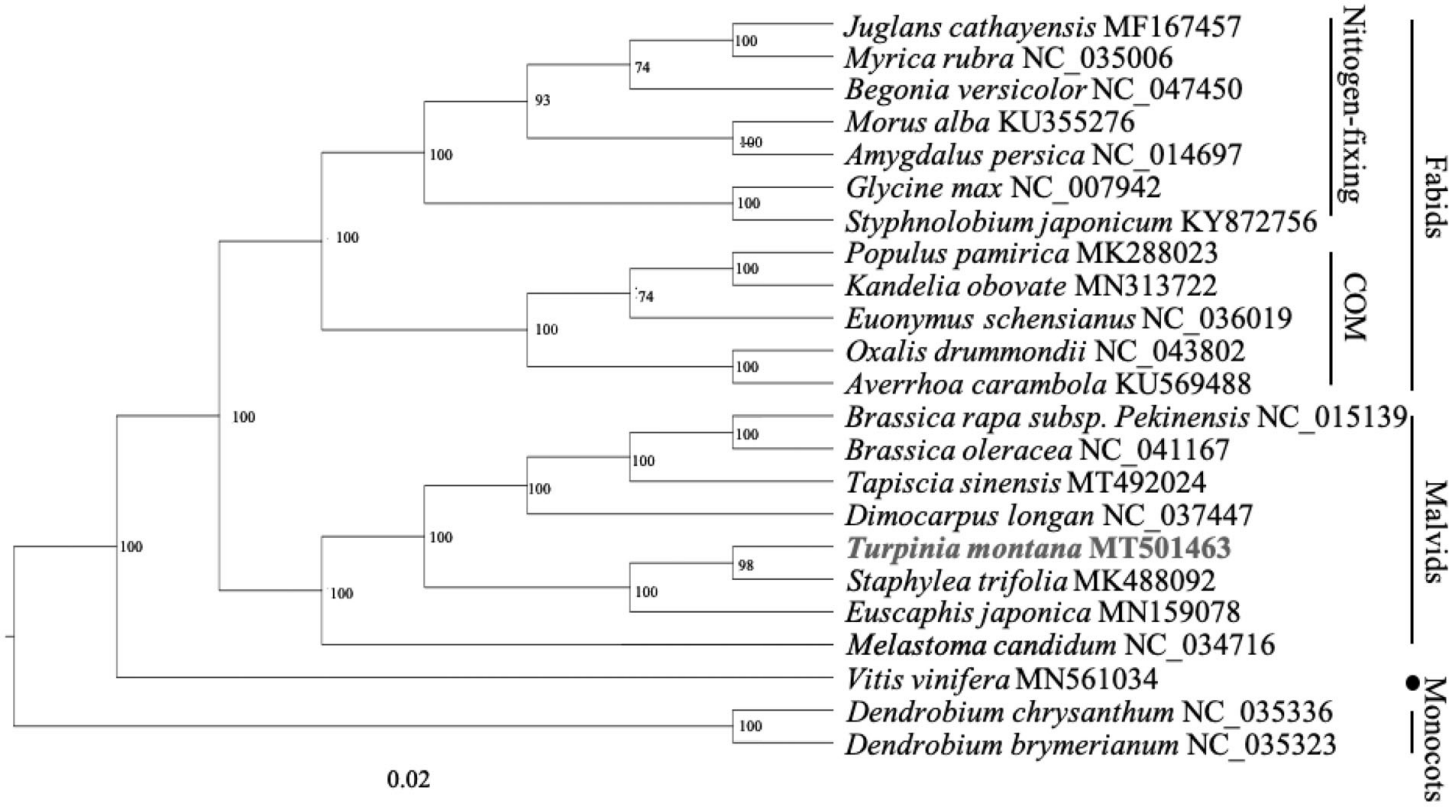
The phylogenetic tree of *Turpinia montana* with other species.

## Data Availability

The data that support the findings of this study are openly available in GenBank of NCBI at https://www.ncbi.nlm.nih.gov, reference number MT501463.
